# Time Slices: What Is the Duration of a Percept?

**DOI:** 10.1371/journal.pbio.1002433

**Published:** 2016-04-12

**Authors:** Michael H. Herzog, Thomas Kammer, Frank Scharnowski

**Affiliations:** 1 Laboratory of Psychophysics, Brain Mind Institute, Ecole Polytechnique Fédérale de Lausanne (EPFL), Lausanne, Switzerland; 2 Department of Psychiatry, University of Ulm, Ulm, Germany; 3 Department of Psychiatry, Psychotherapy and Psychosomatics, Psychiatric Hospital, University of Zürich, Zürich, Switzerland; 4 Neuroscience Center Zürich, University of Zürich and Swiss Federal Institute of Technology, Zürich, Switzerland; 5 Zürich Center for Integrative Human Physiology (ZIHP), University of Zürich, Zürich, Switzerland

## Abstract

We experience the world as a seamless stream of percepts. However, intriguing illusions and recent experiments suggest that the world is not continuously translated into conscious perception. Instead, perception seems to operate in a discrete manner, just like movies appear continuous although they consist of discrete images. To explain how the temporal resolution of human vision can be fast compared to sluggish conscious perception, we propose a novel conceptual framework in which features of objects, such as their color, are quasi-continuously and unconsciously analyzed with high temporal resolution. Like other features, temporal features, such as duration, are coded as quantitative labels. When unconscious processing is “completed,” all features are simultaneously rendered conscious at discrete moments in time, sometimes even hundreds of milliseconds after stimuli were presented.

## Abstract 2

Although we experience the world as a seamless flow of percepts, illusions and experiments suggest that perception operates in a discrete manner. This Unsolved Mystery presents a novel theoretical framework to explain how discrete neural processes create the illusion of a continuous stream of conscious perception.

## Introduction

A diver is jumping from a cliff. We see his trajectory against the blue sky at each single moment in time. Consciousness seems to be a continuous stream of ever smoothly changing percepts, and thus it is intuitively appealing to assume that sensory information is continuously translated into conscious perception. However, already in the third century B.C., the Abhidharma Buddhist school proposed that perception is actually a series of discrete consciousness moments [1]. In Western science, Karl Ernst von Baer coined the term “moment” as the border between the past and the future in the 19th century and, thus, introduced the controversial discrete theories of perception [2]. Discrete theories postulate that conscious perception is a series of distinct moments, similar to snapshots recorded by a video camera [3–8].

Continuous and discrete theories are two opposing poles, and various accounts of intermediate stance have been proposed (for an overview see [9]). All these accounts aim to explain how we can be directly aware of temporally extended events like movement and change, even though our conscious experience is confined to the moment [10]. After all, we are aware of the redness of a red patch as directly as we are of the motion of a moving object, even though color is a “static” visual feature whereas motion requires integration over time [11]. Some philosophers have argued that we can perceive motion and change because our episodes of conscious experience are not momentary, but they extend over time [12,13]. Others avoid this issue by denying the sheer existence of phenomenal consciousness [14,15]. Today, this debate is still unresolved and puzzles philosophers, psychologists, biologists, and neuroscientists alike [3,9,16,17]. Here, we will first review experimental evidence that challenges our intuitive assumption that the world is processed as a seamless stream of ongoing perception. Based on these findings, we will then propose a brain-based framework that describes how time-consuming unconscious processes can give rise to perceptual moments that contain nonstatic temporal information.

Experimental evidence supporting the view that perception might be discrete comes from psychophysical experiments showing that when two stimuli are presented in rapid succession, they are perceived simultaneously, as if they are occurring within a single perceptual episode, i.e., within one snapshot [18–20]. However, the inability to make simultaneity versus nonsimultaneity judgments has also been explained in terms of stimulus persistence at the early levels of the visual system, an interpretation consistent with online continuous perception [21]. Further supporting evidence for discrete theories includes the real-life wagon wheel illusion, where turning wheels can appear to be rotating backwards, as it is argued, due to discrete sampling [7,22]. Conversely, the wagon wheel illusion can also be interpreted by perceptual rivalry without referring to discrete snapshots [23]. Another conversely discussed phenomena is the so-called flash lag illusion: when a moving object and a flash are presented at the same location, the flash is perceived as lagging behind [24]. One possible explanation suggests that the flash resets motion integration, and that motion is newly calculated and postdicted to the time of the flash [17]. According to this view (which is disputed [25]), visual awareness is postdictive and thus seemingly incompatible with continuous theories.

**Fig 1:**
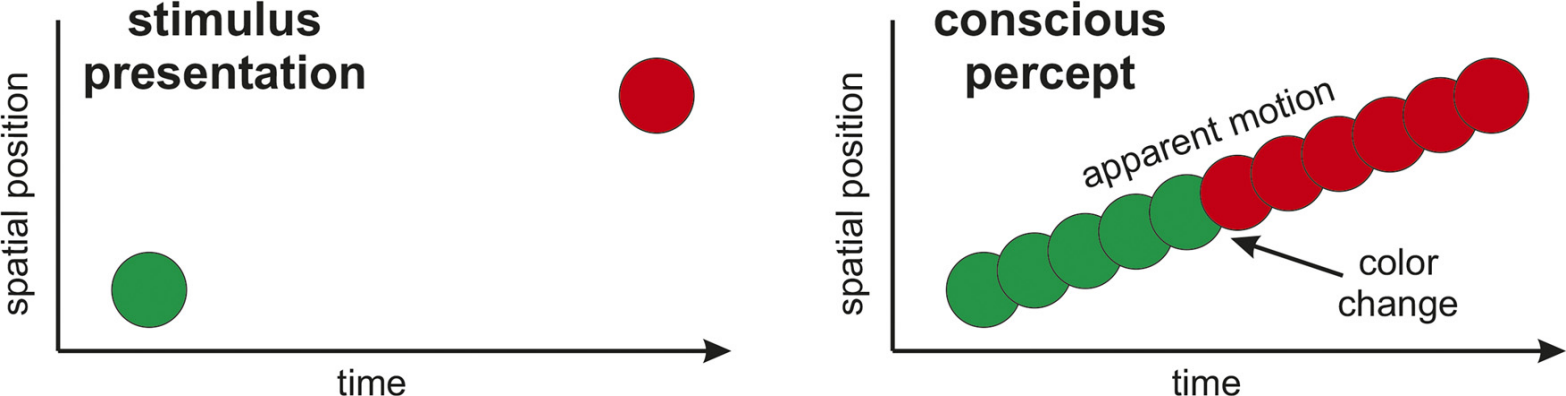
The color phi phenomenon. If two differently colored disks are shown at different locations in rapid succession, observers experience just one disk moving from the first to the second location, changing color abruptly midway on the illusory path. How can the observer know in advance what color the second disk will be and where it will appear? The conscious percept cannot have been formed in a time-ordered fashion, but must have been constructed retrospectively.

One of the most striking examples against continuous theories is the color phi phenomenon [26]. When two colored disks are presented spatially displaced in rapid succession, it appears as if one disk moves between the two positions and changes color in the middle of its trajectory (Fig 1). Logically it is impossible to experience the color change before having seen the second disk. The conscious percept must have been formed retrospectively, thus contradicting continuous theories. As an extreme example, under certain conditions, a stimulus that had been presented first can even be perceived occurring after a stimulus presented later in time [27]. Clearly, such temporal reversals are incompatible with continuous visual perception.

**Fig 2:**
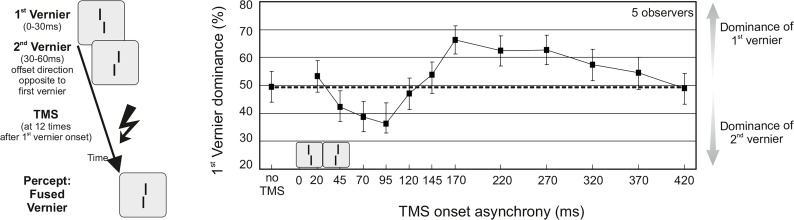
Long-lasting visual feature integration. Feature fusion: A vernier, which is a pair of vertical bars that are spatially offset either to the left or right, is presented in rapid succession with a vernier of opposite offset direction. Interestingly, the two verniers are not perceived individually, one after the other, but as one fused vernier. The perceived spatial offset of the fused vernier is a combination of the offsets of both verniers, i.e., the offsets integrate unconsciously. TMS applied over the occipital cortex at different times after the presentation of the verniers selectively rendered the offset direction of either the first or the second vernier more dominant, even when applied up to 400 ms after the presentation of the verniers. Hence, the integration of the vernier offsets is not completed beforehand, and a conscious percept is only elicited a few hundred milliseconds after stimulus presentation. Vernier presentations are indicated by the small depictions in the graph. Before TMS application, vernier offsets were adjusted such that performance was at 50%, i.e., on average observers reported equally often the offset direction of the first and the second vernier (dashed line). A first vernier dominance of more than 50% indicates that the offset direction of the first vernier was reported more often. Conversely, a first vernier dominance of less than 50% indicates dominance of the second vernier. Adapted from [29].

A more recent study, combining feature fusion and transcranial magnetic stimulation (TMS), showed for the first time that unconscious processing, that precedes the formation of a conscious event, can be quite long lasting. In feature fusion, visual stimuli are presented in rapid succession at the same location rather than at different ones as in the color phi illusion. Because of the short durations, the two stimuli are not perceived individually, but as one fused percept. For example, when a red disk is immediately followed by a green disk, the two disks fuse and are consciously perceived as only one yellow disc. Feature fusion also occurs in acoustics [28], in somato-sensation [28], and with other visual stimuli such as verniers (Fig 2). As with the colored disks, observers cannot tell the two verniers apart. Only the result of their unconscious integration is consciously perceived. Still, TMS can modulate the unconscious integration for up to 400 ms [29]. Hence, under the conditions of this experiment, integration cannot be completed beforehand and consciousness cannot occur before 400 ms. Interestingly, the actual presentation of the two verniers took only 60 ms altogether, i.e., unconscious integration outlasted their presentation by a factor of ~7.

Examples against continuous theories are not limited to vision. The cutaneous rabbit effect is a somatosensory illusion that arises after rapid and successive stimulation of first the wrist, and then of near the elbow [30]. This stimulation creates the illusion of touch along the length of the arm (as though a rabbit hopped along it), even though it is impossible to experience the movement trajectory before having been stimulated near the elbow. Also, comprehension in speech perception is not continuous but can be delayed. For example, in the sentences “the mouse was broken” and “the mouse was dead,” the last word determines the meaning of the noun (i.e., either the computer mouse or the animal). Nevertheless, we do not consciously retroactively edit our interpretation of the word “mouse,” but consciousness is delayed until the meaning of the sentence has been established [31].

All these findings argue clearly against continuous theories of perception. However, they argue also against simple snapshot theories, in which the brain collects visual information only at certain discrete points in time, like a camera. For example, we can perceive apparent motion with differences between visual stimuli of only 3 ms [32], but snapshot models, which sample, say, every 40 ms, would simply miss the second stimulus. Likewise, with a high sampling rate we cannot explain why two disks displayed with ~40 ms delay are perceived as occurring within one moment of time [20]. A high sampling rate is also incompatible with the feature fusion experiment (Fig 2), in which conscious perception can be delayed for several hundred milliseconds [29].

How can these contradictory findings and ideas be reconciled? We propose a two-stage model, in which first visual information is unconsciously processed with relatively high temporal resolution (Box 1). Second, conscious percepts occur at a much slower rate, at discrete moments, “representing” the output of unconscious processing.

**Box 1. Temporal Resolution of Sensory Systems**

The temporal resolution of sensory systems depends on several factors. First, the physical signal of a sensory stimulus needs to be converted into a brain signal. The visual system relies on electrochemical transduction (i.e., light energy causes a change in the protein rhodopsin in the retina), whereas in the auditory and somatosensory systems, sound vibrations or touch are mechanically converted into electrical signals. Second, the signal within the sensory system has to be processed such that changes between stimuli can be detected. Three general types of temporal resolution can be distinguished: (a) the ability to discriminate the repetition of a periodical signal, e.g., a flickering light or fluttering sound (same system, same channel); (b) the ability to discriminate the onsets of two signals applied to two different channels within a sensory system, e.g., beeps to the left and right ears or two flashes at different visual sites (same system, different channels); and (c) the ability to discriminate the onsets of two signals applied to two different sensory systems (different systems, different channels).

(a) Our sensory systems differ remarkably in terms of the temporal scale along which they can detect the difference between two stimuli. Whereas two clicks can already be separated if they are only 1–3 ms apart [33], two taps need to be about 10 ms apart [33], and two flashes about 25 ms [34]. However, for trains of stimuli, the presentation rate at which the sensation of flicker ceases is similar for the visual and auditory systems, i.e., at around 16 ms inter-stimulus-interval (ISI) [35,36].
(b) Temporal order judgement within a sensory system is in the range of 20–50 ms ISI and has been found not to differ substantially between the modalities [37–39]. However, under particular circumstances the auditory system yields faster discrimination compared to the visual system [40].
(c) Comparing the order of events across different sensory systems has approximately the same temporal resolution as within a sensory system [37]. This has been taken as evidence for a supra-sensory timing mechanism that is involved in the detection of onsets across all modalities [41]. However, the lack of transfer of learned temporal order judgements between modalities questions the existence of a supra-sensory timing mechanism [40].

## The Two-Stage Model

**Fig 3:**
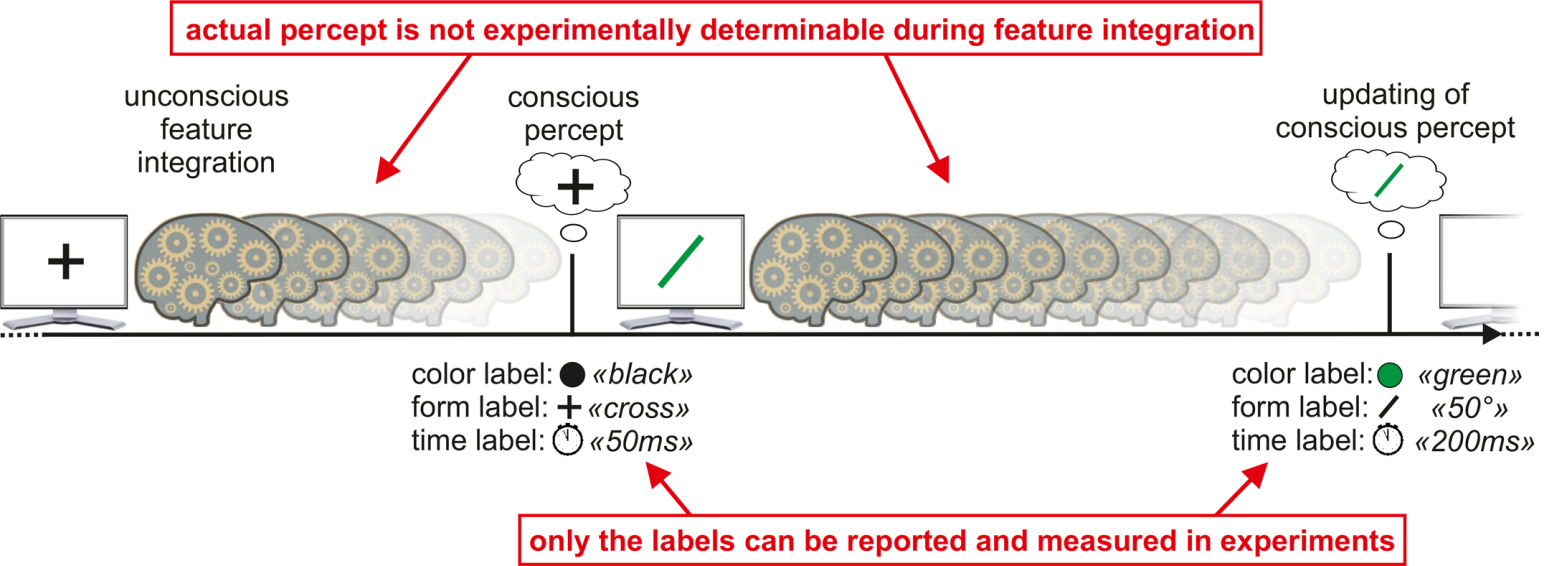
Two-stage model of visual perception. A stimulus, for example, a cross, is presented on a computer screen. Sensory information about the cross is quasi-continuously and unconsciously analyzed by feature detectors. Just like color and orientation, temporal features are also coded as quantitative labels. When unconscious feature integration is completed, all features are simultaneously rendered conscious at one discrete point in time, sometimes even hundreds of milliseconds after the object was presented [29]. The conscious representation can be seen as a feature vector, which contains a value that represents the stimulus best for each feature dimension, for example, a black cross with a 50 ms duration. Only the feature labels can be reported and measured in experiments, but the actual percept during feature integration is not experimentally determinable. It might be that the previously generated conscious percept stays constant until the next percept is elicited, or that the percept may be rendered conscious just for a moment of time and there is no perception until the next percept emerges (as shown here).

According to our model, the elements of a visual scene are first unconsciously analyzed. This period can last up to 400 ms and involves, amongst other processes, the analysis of stimulus features such as the orientation or color of elements and temporal features such as object duration and object simultaneity. Just like a color detector “labels” the stimulus shown in the example in Fig 3 as “green,” and an orientation detector labels it as “oblique,” a duration detector assigns the label “50 ms.” Upon completion of the analysis, the features are integrated into a coherent, conscious percept. All features become conscious simultaneously, and the percept contains all the feature information derived from the various detectors. Hence, the green line in Fig 3 is not actually consciously perceived as green during its actual presentation but later when rendered conscious. The same holds true for temporal features. The stimulus is not perceived during the 50 ms when it is presented. The stimulus is even not perceived for a duration of 50 ms. Its duration is just encoded as a “number,” signifying that the duration was 50 ms in the same way that the color is of a specific hue and saturation.

According to our model, the feature detectors are operating quasi-continuously and can thus analyze features with a high temporal resolution compared to the conscious percepts, which are generated at a much lower rate (Box 2). Visual information processing is similar to a sample and hold mechanism in engineering as they are frequently used, for example, in analog/digital converters. As a simple example, consider a digital watch. Invisible to the beholder, the watch tracks the actual time with high temporal resolution by counting the oscillations of a vibrating quartz. After a predetermined number of oscillations, the digital display changes from, say, 23 hours and 7 minutes to 23 hours and 8 minutes. The display remains in this state for the next minute, similarly to the conscious percept, which stays constant until the next percept is elicited.

**Box 2. Low Pass Filtering**

A common argument against discrete theories is based on low pass filtering mechanisms, for example, based on mathematical convolution. The basic idea is that sensory information enters a buffer where it is integrated with preceding and following information. Like the input, the output is also continuous [42]. For example, in the feature fusion experiment, the sensory information of both verniers is integrated; therefore, conscious perception is delayed.

However, because such approaches assume that perception is continuous, humans would perceive first the first vernier offset, then a mixture of the two, and then the second vernier offset. In reality though, this is not the case. We perceive only one, static, fused vernier, which does not change over time. Another problem is that integration in the feature fusion experiment lasts very long (i.e., 400 ms), whereas integration needs to be much shorter in apparent motion paradigms, in which we can perceive differences of only 3 ms. Low pass filtering mechanisms thus suffer from the same problem as basic snapshot models.

Our model avoids these problems by decoupling sensory processing and perception, as well as their respective time courses. However, also in our model, (unconscious) processing occurs in a buffer and (conscious) perception is delayed. The difference to low pass filtering is that, according to our model, the output of unconscious processing is discrete, meaningful, and rendered conscious at once. Large parts of unconscious processing will never reach consciousness.

We would like to mention that, contrary to the watch example, we are not suggesting that conscious percepts occur periodically and have identical durations. Instead, we propose that a percept is elicited when unconscious processing reaches an attractor state [43,44]. The trajectory towards the attractor state remains unconscious. Only attractor states code for the things we perceive, whereas non-attractor states never reach consciousness. It will be important to show how attractor and non-attractor states are coded on the neural level.

Our two-stage model is, first, not a standard snapshot model, in which pictures of the world are taken at discrete times. According to our model, snapshots are of ongoing, quasi-continuous, unconscious processing (see also [16]). Second, the snapshots are not randomly taken at arbitrary times of unconscious processing. Instead, we propose that the snapshots represent integrated, meaningful outputs of unconscious brain processing, namely, attractor states. The snapshots are like a feature vector that contains a value for each relevant feature dimension of a stimulus, which together, constitute a meaningful post-hoc representation of the event that occurred during the unconscious processing period (Fig 3). Metaphorically, such a representation is akin to the answer to the question of how were your holidays: “We enjoyed the colors of the Tuscan landscape for three days and then went to Venice for four sunny days at the sea.” The response is a compressed post-hoc description regarding the temporal features of the trip even though the actual event was spread over a long period of time.

Why do we not see sudden changes between the discrete percepts, as in a low temporal resolution movie? As in any system, we cannot go beyond its resolution, i.e., we perceive time as continuous just as we perceive a line as continuous even though its ink is of discrete atomic nature.

## Time-Consuming Unconscious Feature Integration

Why is such a long period of unconscious feature integration needed when our feature detectors can operate much faster? Vision involves complex and ill-posed problems, which can only be solved in a recurrent and time consuming manner [45,46]. This is particularly evident in the case of motion. Take a football game as an example. Twenty-two players are constantly moving around, all following their own trajectory. First, just to compute one motion trajectory, the brain needs to integrate information across time and space. Hence instantaneous perception is impossible. Second, the 2-D projections of the players on the retina often cross. In principle, there are infinitely many possible 3-D world scenarios that can give rise to the very same 2-D retinal projection. To compute the best out of a potentially infinite number of options in a recursive manner, the brain needs time. For this reason, the brain functions such that we consciously perceive only the most plausible solution, and not a confusing manifold of possibilities that occur during unconscious processing. The unconscious feature integration period is the period of sense-making.

As mentioned, percepts occur when processing has converged to an attractor state. The duration of convergence depends on the complexity of the processing. Feature integration does not always require lengthy computations. For example, fight-or-flight reflexes can be very fast, and motor responses can occur before a conscious percept is fully developed. In these cases, action is even faster than perception, i.e., actions can be elicited by unconscious processing alone.

## Discussion

We experience the world as a continuous stream of smoothly changing percepts. However, past and recent psychophysical evidence suggest that perception is discrete. Nevertheless, simple snapshot theories quickly run into problems because they, for example, cannot explain the relatively high temporal resolution of the visual system compared to slow conscious perception. For this reason, we propose a two-stage model that decouples quasi-continuous unconscious feature processing from conscious percepts, which occur only at discrete moments in time. The conscious percept represents the output of unconscious processing, which has relatively high temporal resolution. Unconscious processing evolves over time, whereas conscious perception is discrete.

The most important implication of our model is that the duration of a conscious percept and the temporal resolution of the visual system are independent issues. Thus, investigating the former does not allow us to draw conclusions regarding the latter. As mentioned, experiments have shown that humans perceive two disks as simultaneous when they are presented within ~40 ms of one another [20]. This result was taken as evidence that perception lasts at least 40 ms. However, the experiment tells us only that the temporal resolution of *unconscious* processing is limited in this paradigm. No conclusions can be drawn about the duration of the percepts. Likewise, reaction times in many experiments show periodic variations and were often taken as evidence for discrete theories, in which information processing depends on brain oscillations [16,47]. In a similar vein, the wagon wheel illusion seems to speak to such brain oscillations [7]. We, to the contrary, argue that these experiments may reveal periodic unconscious processes but cannot be linked to conscious perception. To investigate the minimal duration of a conscious percept, we need to rely on indirect measures such as the feature fusion paradigm combined with TMS [29]. This experiment revealed that feature fusion is not completed before 400 ms, and consequently consciousness cannot occur beforehand. It thus provides a lower bound for the minimal duration of percepts in this paradigm. However, this experiment does not provide an upper bound and cannot directly measure the duration of percepts. Other experimental methods with high temporal resolution, such as electroencephalography (EEG) (e.g., [48]) and especially electrophysiology (e.g., [49]), have provided valuable insights into the temporal dynamics of unconscious processing by providing objective markers of temporal processing. Complementarily, functional magnetic resonance imaging (fMRI) work has been successfully used to identify the spatial locations of neural structures involved in the unconscious processing of time information (e.g., [50]).

According to the two-stage model, we do not experience stimuli and objects during their actual presentation, but much later, when they are rendered conscious. For example, a 50 ms stimulus is not perceived for 50 ms during its presentation, but its perceived duration is the result of unconscious processing that assigns a quantitative duration label to this stimulus [51–54]. This is akin to how, for example, motion features are processed. We do not perceive motion because we consciously track a dot point by point along its trajectory, but because the output of motion detectors provides quantitative values for direction and speed. Hence, psychophysical paradigms cannot reveal what we actually experience during feature integration but can only reveal the consciously perceived output of processing, i.e., the feature labels (Fig 3). Furthermore, the coding of temporal features as quantitative labels can explain the long-standing philosophical mystery regarding how we can experience nonstatic temporal features even though our conscious experience is confined to the moment [9–13].

With our model, temporal illusions such as apparent motion, the color-phi phenomenon, feature fusion, the flash lag effect, and many more can easily be explained. During the unconscious processing period, the brain collects information to solve the ill-posed problems of vision, for example, using Bayesian priors [55–59]. The percept is the best explanation in accordance with the priors given the input. For example, in the color-phi phenomenon, object constancy is more plausible than the abrupt temporal disk onsets and offsets, and thus the two disks are interpreted as one moving disk whose color changes. Temporal aspects of a stimulus are balanced with other priors, such as object constancy, proximity grouping, contextual expectations, etc. For example, temporal reversals (i.e., when a first-presented stimulus is perceived as being presented later in time) occur because the temporal order information is reduced so much (by reducing the stimulus onset asynchrony and by varying the contrast), that it becomes overruled by the saliency cue, which usually favors later stimuli (as demonstrated, for example, by the recency effect in memory [60], and by backward masking being stronger than forward masking [27,61]). Also contributing are other unconscious processes, such as spatially localized changes in the temporal impulse response of early visual neurons. For example dark adaptation decreases the firing threshold and thus might prolong the integration period, which can explain why perceived duration can change as a function of the context in which the stimuli are presented [62–64]. In our model, perceptual illusions lose part of their mystery because perception is, to a certain extent, decoupled from stimulus presentation and processing.

One important question is how the brain “knows” when unconscious processing is complete and can be rendered conscious. We speculate that percepts occur when processing has converged to an attractor state [43,44]. One possibility is that hitting an attractor state leads to a signal that renders the content conscious, similarly to, for example, broadcasting in the global workspace theory [65]. Another option is that attractor states are the conscious states. To shed light on these speculations, future research needs to provide more insights into what distinguishes conscious from unconscious states. Related questions are the role of cognition, volition, and attention in these processes. We speculate that these can strongly bias unconscious processing towards specific attractor states. For example, when viewing ambiguous figures, a verbal hint or shifting attention can bias observers to perceive either one of the possible interpretations, each corresponding to a different attractor state [66].

We suggest that mechanisms similar to the ones related to the minimal time of a percept also operate on longer time scales [67,68]. For example, full comprehension of a sentence sometimes requires waiting until the last word. The sentences, "The mouse was broken," and "The mouse was dead," differ radically in meaning, but this difference is determined only by the last word [31]. Real-time understanding would have to be fragmented since the last word needs to be integrated with the word "mouse," which can take substantial time depending on the speed of reading or speaking.

As mentioned, stimuli presented within ~40 ms are often perceived as occurring simultaneously [20]. In schizophrenic patients, the integration window is much longer [69]. Interestingly, schizophrenic patients often report that the stream of perception is strongly fragmented. In this respect, patient studies may offer important insights into the structure of temporal processing.

Our considerations go well beyond perception research. They are also crucial for neuroscience and computer vision, which both have to provide answers to the question of what aspects of processing are rendered “conscious,” and at what time. Our model also advances neural coding theory because it allows distinctions to be made between neural states corresponding to the unsconsious processing and stable attractor states that represent the meaningful percept. As importantly, our model challenges prominent theories on philosophy of mind, which assume that consciousness is a continuous stream.

## Abbreviations

EEG: electroencephalography
fMRI: functional magnetic resonance imaging
ISI: inter-stimulus-interval
TMS: transcranial magnetic stimulation
