# Correction: Time Slices: What Is the Duration of a Percept?

**DOI:** 10.1371/journal.pbio.1002493

**Published:** 2016-06-07

**Authors:** The PLOS Biology Staff

## Notice of Republication

This article was republished on May 19^th^, 2016 to correct poor figure quality in the PDF version, which was introduced during the typesetting process. The publisher apologizes for these issues with the figure quality. Please download this article again to view the updated version.

## References

[1] Herzog MH, Kammer T, Scharnowski F (2016) Time Slices: What Is the Duration of a Percept? PLoS Biol 14(4): e1002433 doi: 10.1371/journal.pbio.1002433 27070777 10.1371/journal.pbio.1002433 PMC4829156

